# Factors associated with unplanned readmissions for patients with mental and behavioural disorders in China: a quantitative analysis

**DOI:** 10.1080/16549716.2024.2435642

**Published:** 2025-01-20

**Authors:** Sha Lai, Zechen Wang, Chi Shen, Junfei Feng, Yawei Huang, Xiaolong Zhang, Li Lu, Zhongliang Zhou

**Affiliations:** a Health Management and Policy Institute, School of Public Policy and Administration, Xi’an Jiaotong University, Xi’an, China; b System Behavior and Management Laboratory, Philosophy and Social Sciences Laboratory of the Ministry of Education, Xi’an Jiaotong University, Xi’an, China

**Keywords:** Unplanned readmission, mental and behavioural disorders, risk factors, health outcomes, rehospitalisation

## Abstract

**Background:**

Unplanned readmissions among patients with mental and behavioural disorders (MBDs) disrupt inpatient recovery and impose financial burdens on families and healthcare systems.

**Objectives:**

To estimate the 31-day unplanned inpatient readmission rates for MBDs in China and identify determinant profiles from the perspective of individual, hospital, and contextual levels.

**Methods:**

Data from patients with MBDs were collected from the medical records of 99 public hospitals across 10 cities. A total of 49,352 inpatient admissions were analysed based on the proposed conceptual model using multilevel logistic regressions.

**Results:**

The 31-day unplanned readmission rate (excluding 0–1-day returns) was 8.6% (95% CI: 8.4–8.9%). Determinant profiles differed across the overall group and subgroups. The number of general practitioners within cities was associated with reduced risk of unplanned readmissions. Hospital factors such as facility type and size, human resources, and revenue size were associated with unplanned readmissions only in specific subgroups. Additionally, individual-level factors, including demographic information (e.g. gender, age, marital status, and occupational status), disease-related factors (e.g. primary diagnostic group, condition at admission, and other diagnoses), and clinical characteristics (e.g. length of stay and medical costs), were associated with unplanned readmissions across all subgroups.

**Conclusion:**

The study emphasises collaborative efforts from health systems, hospitals, and patients to reduce unplanned readmissions for MDBs. Health systems should focus on improving access to care, enhancing quality, and ensuring continuity while providing incentives for hospitals. Additionally, hospitals should prioritise the identification and effective management of their high-risk patients.

## Paper context

- **Main Findings:** Contextual health resources and high-quality health providers can protect against hospital readmissions.
- **Added Knowledge:** Readmissions among patients with mental and behavioural disorders are a systemic issue associated with sociodemographic, disease, clinical, healthcare providers, and resource availability factors.
- **Global health impact for policy and action:** The worldwide prevalence of mental disorders underscores the urgent need to strengthen the longitudinal continuity of primary care and improve the quality of mental health services.

## Background

Unplanned readmission (also termed ‘unplanned hospital readmission’ [1]) refers to an unexpected subsequent or repeat hospitalisation after an index admission under specific conditions [2]. Unplanned readmission is prevalent, resource-intensive, and exerts adverse effects on both patients and healthcare systems. Policymakers in Denmark, England, Germany, and the United States have contemplated strategies to reduce readmissions [3]. In China, unplanned readmission is a relatively new phenomenon and has gained attention from health authorities and researchers [4]. According to a survey of China’s medical service and quality safety data from 2017 to 2020, the 31-day unplanned readmission rate (URR) at secondary and higher public hospitals was 2.92% in 2019 [4]. The URR is a key performance indicator in health services optimisation, as outlined in the Healthy China 2030 Plan. The URR for mental illness-related hospitalisation ranges from 4.5% [5] to 43.3% [6]; in contrast, the URR for general health conditions ranges from 2.8% to 38% [1,7]. Globally, health services face challenges associated with high costs, low quality, and limited access to mental health care. For patients with mental and behavioural disorders (MBDs), readmission immediately after discharge disrupts short-term recovery, negatively affects long-term outcomes, and leads to dependence on psychiatric services [8,9].

Most studies from high-income countries or regions, such as the United States, have emphasised individual-level risk and protective factors, including sociodemographic factors [10–13], health conditions [14], principal discharge diagnosis, and clinical factors [13,15], for psychiatric readmissions. Fewer studies have addressed the structural- or institutional-level challenges [16]. A systematic review of unplanned readmissions for MBDs identified lower levels of education, unemployment, previous psychiatric hospitalisations, and hospitalisation longer than 7 days as the risk factors for unplanned readmissions [1]. Empirical evidence from patient and caregiver surveys has elucidated the effects of delayed initial psychiatric admissions [17], involuntary admissions [18], patient compliance with follow-up, outpatient visits and treatment [19–21], and the role of peer support workers [22] on unplanned readmissions. A few studies have explored the influence of policy-related and environmental factors on unplanned readmissions using cross-time or cross-regional comparisons [23]. Hospitals may encourage patient readmissions for financial gain, particularly when incentivised by government policies that encourage increased admissions to improve revenue or shorten the length of hospitalisation (e.g. diagnosis-related group-based payments) [24]. Clinical treatments substantially affect readmission rates [25], but this aspect was beyond the primary focus of this study.

Evidence-based interventions from hospitals, communities, and macro-health policies can prevent up to 9–59% of unplanned readmissions [3,26,27]. In mainland China, few studies have analysed the risk factors for unplanned readmissions for psychiatric disorders [28]. This study aims to address the gap in understanding hospital and contextual factors associated with readmissions in order to allow a more systematic analysis of the issue. Moreover, since most existing literature originates from high-income countries or areas, it is essential to extend research to more diverse regions to account for the influence of different health systems on readmissions.

## Conceptual framework

**Figure 1.**
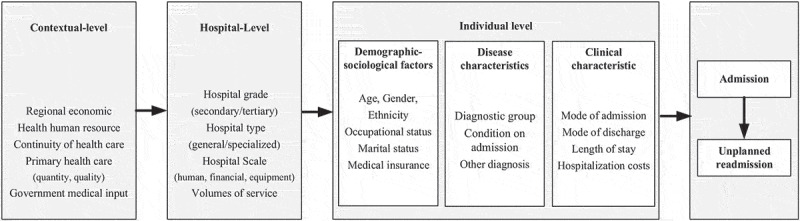
Conceptual framework of factors associated with unplanned readmission among patients with mental and behavioural disorders.

Based on a literature review [29,30] and expert consultations to identify the factors influencing the URR in patients with MBDs, we constructed a conceptual model (Figure 1). Andersen’s behavioural model [31] postulates that a patient’s social, behavioural, and environmental surroundings can impact health outcomes. The model has been previously used as an explanatory framework for readmission [32]. The conceptual model encompassed individual, hospital, and contextual factors that are easily measured and may impact on post-discharge outcomes.

Hospital admissions are primarily influenced by demographic characteristics and healthcare needs [31]. Hospital-level factors, e.g. hospital grade and type, human resource scale, and volume of services, directly impact the quality of medical care. Contextual factors encompass the social environment (regional economy), human health resources, continuity of care (downward referral rates), primary health care (e.g. the number of general practitioners and family doctor contract rates) and government subsidies for public hospitals, all of which affect health services. The availability of regional health resources and the organisation of healthcare systems determine health service utilisation [31,33,34]. Existing predictive models on hospital readmission focus on sociodemographic, disease, and clinical characteristics at the individual level but rarely encompass hospital and contextual factors [1]. This study aimed to address this research gap by including data from multiple sources on unplanned readmissions among patients with MBDs.

## Methods

### Data collection and study sample

This retrospective study consisted of data from hospitals in Y Province, western China, that met the eligibility criteria. The inclusion criteria were as follows: (i) secondary and tertiary general public hospitals with psychiatric inpatient services; (ii) secondary and tertiary public specialised psychiatric hospitals (*n* = 105). The exclusion criteria were as follows: hospitals with data exchange and quality deficiencies in electronic medical records. Six hospitals were excluded. Finally, data from 99 public hospitals, comprising 91 general hospitals and eight specialised psychiatric hospitals, were analysed. Y Province, comprising 39.6 million residents in 2023, ranks at a mid-level among 31 provincial regions for population and socioeconomic development. To maintain confidentiality, the province name is not provided herein.

Data were extracted from the front pages of medical records from 2021 to 2022 for all individuals admitted with a primary diagnosis of MBD based on the International Classification of Diseases-Tenth Revision (ICD-10) (codes F00–F99). Death cases were excluded. Data included 50,230 hospital discharge records from non-deceased patients. Because of missing data on major explanatory variables, 1.75% of the cases were excluded. Finally, data from 49,352 hospital discharges were analysed, with multiple records per patient suggesting readmissions.

Based on the conceptual framework, hospital profiles and contextual information data were collected from prefecture-level cities. Hospital data were obtained from administrative records, whereas contextual data for 10 prefecture-level cities were obtained from healthcare administrative records and statistical yearbooks of Y Province.

This study used pre-existing, de-identified, and anonymised data to protect the privacy of patients, healthcare professionals, and hospitals. The protocol was approved by the Research Ethics Committee of Xi’an Jiaotong University. Additionally, institutional review boards of all participating hospitals approved the study. The need for informed consent was waived because of data anonymisation.

## Variables

### Outcome variables

Unplanned readmissions to the same hospital within 2–31 days is the primary outcome. Risk factors for unplanned readmission were based on the time interval, with early readmissions – typically within 2–31 days – potentially indicating problems in care coordination or incomplete discharge planning [20,35]. However, readmissions after 1 month may be attributed to illness severity [36]. A 31-day interval was used for consistency with medical records and policy documents.

According to Landrum et al. [2], the readmission criteria were as follows: (i) identifying hospital admissions through a patient identifier to distinguish index from subsequent admissions; (ii) establishing a clinical diagnosis, with readmission defined as a return to the same hospital with the same major diagnostic category (i.e. MBDs, ICD-10 codes F00–F99); (iii) purpose of readmission, with disease recurrence or exacerbation within the same diagnostic category considered unplanned readmission; planned readmissions were excluded per the 31-day criteria documented on the front pages; and (iv) discharge-toreadmission interval, utilising the 31-day interval based on the Chinese health policy guidelines. Similar to other studies [37], unplanned readmissions within days zero to one were excluded from the regression analyses because of correlations with split or interrupted hospitalisation [37]. Additionally, 60- and 90-day intervals were used to calculate the URR. Patients discharged within the last 31, 60, or 90 days of the study were excluded to ensure an appropriate follow-up duration. A length of stay (LOS) < 24 h was not considered an inpatient hospitalisation [2,28].

In the regression analyses, unplanned readmission was considered a binary outcome of a previous admission, coded as zero (no unplanned readmission within 2–31 days) or one (with unplanned readmission) after discharge.

### Explanatory variables

Based on the previous literature and the conceptual framework, numerous individual, hospital, and contextual factors were selected as explanatory variables. Details on the definitions and measurements of the explanatory variables are provided in Appendix Table S1.

Primary diagnosis was categorised into the following four groups according to the ICD-10 [38]: (i) schizophrenia, schizotypal, and delusional disorders (hereafter termed ‘schizophrenia’ for brevity, ICD-10 codes F20–F29, *n* = 15,270, 30.94%); (ii) affective disorders (ICD-10 codes F30–F39, *n* = 11,299, 22.89%); (iii) neurotic, stress-related, and somatoform disorders (hereafter termed ‘neuroticism’ for brevity, ICD-10 codes F40–F48, *n* = 11,452, 23.20%); and (iv) other MBDs not included in these three categories (*n* = 11,331, 22.96%).

### Data analysis

Multilevel binary logistic regression analyses were conducted, with contextual-level analyses at prefecture city (Level 3), hospital (Level 2), and individual (Level 1) factors, according to the data nesting structure and conceptual framework. The final sample size for the full-sample regression consisted of 49,352 discharges from 99 hospitals across 10 prefecture-level cities. First, null models without explanatory variables were run to quantify the variance attributed to contextual- and hospital-level random effects. Second, three models were used to examine the 31-day unplanned readmission for patients with MBDs. Model 1 utilised individual factors as independent variables. In Model 2, hospital factors were added to the variables in Model 1. In Model 3, contextual factors were added to the variables in Model 2. Clustering effects within patients were adjusted using robust standard errors. Intraclass correlation coefficients were based on multilevel random-intercept logistic regression models. A subgroup analysis of MBDs (schizophrenia, affective disorders, and neuroticism) was conducted to map the disease-specific characteristic profiles.

All data were analysed using Stata version 18.0 (Stata, College Station, TX), with statistical significance set at α = 0.05 (two-tailed).

## Results

### Sample characteristics

Hospital discharge data from 49,352 patients with MBDs (female: 51.10%) were analysed. Specifically, 30.94% (*n* = 15,270) of the patients were diagnosed with schizophrenia, 22.89% (*n* = 11,299) with affective disorders, 23.20% (*n* = 11,452) with neuroticism, and 22.96% (*n* = 11,331) with other conditions. Table 1 summarises the individual, hospital, and contextual characteristics.

**Table 1.** Individual-, hospital-, and contextual-level characteristics of patients with mental and behavioural disorders.

		Overall (n, %) (*n* = 49,352)	Schizophrenia, schizotypal, and delusional disorders (n, %) (*n* = 15,270, 30.94%)	Affective disorders (n, %) (*n* = 11,299, 22.89%)	Neurotic, stress-related and somatoform disorders (n, %) (*n* = 11,452, 23.20%)	Others (n, %) (*n* = 11,331, 22.96%)
**Individual level characteristics**						
Age (years)	0 ~ 17^†^	7,814 (15.83%)	378 (2.48%)	1,657 (14.67%)	528 (4.61%)	5,251 (46.34%)
	18 ~ 40	15,572 (31.55%)	7,255 (47.51%)	4,356 (38.55%)	2,343 (20.46%)	1,618 (14.28%)
	41 ~ 65	19,625(39.77%)	6,819 (44.66%)	4,051 (35.85%)	6,520 (56.93%)	2,235 (19.72%)
	65 or above	6,341(12.85%)	818 (5.36%)	1,235 (10.93%)	2,061 (18.00%)	2,227 (19.65%)
Gender	Male^†^	24,132 (48.90%)	8,603 (56.34%)	4,653 (41.18%)	3,539 (30.90%)	7,337 (64.75%)
	Female	25,220 (51.10%)	6,667 (43.66%)	6,646 (58.82%)	7,913 (69.10%)	3,994 (35.25%)
Ethnicity	Han^†^	48,832 (98.95%)	15,173 (99.36%)	11,168 (98.84%)	11,233 (98.09%)	11,258 (99.36%)
	Non-Han	520 (1.05%)	97 (0.64%)	131 (1.16%)	219 (1.91%)	73 (0.64%)
Occupational status	Employed^†^	24,685 (50.02%)	9,061 (59.34%)	6,025 (53.32%)	6,284 (54.87%)	3,315 (29.26%)
	Unemployed	2,989 (6.06%)	1,595 (10.45%)	700 (6.20%)	447 (3.90%)	247 (2.18%)
	Non-employed	10,405 (21.08%)	2,167 (14.19%)	3,133 (27.73%)	2,168 (18.93%)	2,937 (25.92%)
	Others	11,273 (22.84%)	2,447 (16.02%)	1,441 (12.75%)	2,553 (22.29%)	4,832 (42.64%)
Marital status	Unmarried^†^	18,163 (36.80%)	6,607 (43.27%)	4,173 (36.93%)	1,392 (12.16%)	5,991 (52.87%)
	Married	25,401 (51.47%)	5,882 (38.52%)	6,071 (53.73%)	9,227 (80.57%)	4,221 (37.25%)
	Others	5,788 (11.73%)	2,781 (18.21%)	1,055 (9.34%)	833 (7.27%)	1,119 (9.88%)
Medical insurance	Self-pay^†^	6,212 (12.59%)	1,392 (9.12%)	1,210 (10.71%)	993 (8.67%)	2,617 (23.10%)
	UEBMI	9,968 (20.20%)	2,894 (18.95%)	2,207 (19.53%)	3,365 (29.38%)	1,502 (13.26%)
	URRBMI	23,512 (47.64%)	7,971 (52.20%)	6,950 (61.51%)	5,102 (44.55%)	3,489 (30.79%)
	Others	9,660 (19.57%)	3,013 (19.73%)	932 (8.25%)	1,992 (17.39%)	3,723 (32.86%)
Condition on admission	Symptomatic^†^	44,667 (90.51%)	14,411 (94.37%)	10,703 (94.73%)	8,898 (77.70%)	10,655 (94.03%)
	Clinically undetermined	2,546 (5.16%)	135 (0.88%)	237 (2.10%)	1,724 (15.05%)	450 (3.97%)
	Unknown or no symptom	2,139 (4.33%)	724 (4.74%)	359 (3.18%)	830 (7.25%)	226 (1.99%)
Mode of admission	Emergency^†^	8,642 (17.51%)	2,201 (14.41%)	2,162 (19.13%)	2,437 (21.28%)	1,842 (16.26%)
	Elective admission	32,563 (65.98%)	10,665 (69.84%)	8,776 (77.67%)	7,827 (68.35%)	5,295 (46.73%)
	Others	8,147 (16.51%)	2,404 (15.74%)	361 (3.19%)	1,188 (10.37%)	4,194 (37.01%)
Mode of discharge	Discharge following medical advice^†^	43,689 (88.53%)	12,933 (84.70%)	10,564 (93.49%)	10,252 (89.52%)	9,940 (87.72%)
	Transferred following medical advice	240 (0.49%)	40 (0.26%)	66 (0.58%)	50 (0.44%)	84 (0.74%)
	Discharged against medical advice	1,209 (2.45%)	174 (1.14%)	365 (3.23%)	267 (2.33%)	403 (3.56%)
	Others	4,214 (8.54%)	2,123 (13.90%)	304 (2.69%)	883 (7.71%)	904 (7.98%)
Other diagnoses	No other diagnosis^†^	16,941 (34.33%)	7,036 (46.08%)	4,666 (41.30%)	2,200 (19.21%)	3,039 (26.82%)
	With other psychiatric diagnosis	3,544 (7.18%)	192 (1.26%)	277 (2.45%)	541 (4.72%)	2,534 (22.36%)
	With other non-psychiatric diagnosis	28,867 (58.49%)	8,042 (52.67%)	6,356 (56.25%)	8,711 (76.07%)	5,758 (50.82%)
LOS	0–7 days^†^	9,713 (19.68%)	680 (4.45%)	1,107 (9.80%)	5,504 (48.06%)	2,422 (21.37%)
	8–14 days	8,500 (17.22%)	971 (6.36%)	1,962 (17.36%)	3,177 (27.74%)	2,390 (21.09%)
	15–30 days	13,571 (27.50%)	4,225 (27.67%)	4,668 (41.31%)	1,857 (16.22%)	2,821 (24.90%)
	30 days or above	17,568 (35.60%)	9,394 (61.52%)	3,562 (31.52%)	914 (7.98%)	3,698 (32.64%)
Total medical cost	Low^†^	16,405 (33.24%)	3,310 (21.68%)	3,359 (29.73%)	5,562 (48.57%)	4,174 (36.84%)
	Middle	16,335 (33.10%)	4,680 (30.65%)	4,275 (37.84%)	4,308 (37.62%)	3,072 (27.11%)
	High	16,612 (33.66%)	7,280 (47.68%)	3,665 (32.44%)	1,582 (13.81%)	4,085 (36.05%)
**Hospital level characteristics (*n* = 99)**						
Hospital size		Number of active employees			996 (sd = 84)	
		Total hospital revenue			408,125 (sd = 68,573)	
Hospital type		General hospital^†^			91 (91.92%)	
		Specialized hospital			8 (8.08%)	
Hospital grade		Secondary ^†^			73 (73.74%)	
		Tertiary			26 (26.26%)	
Hospital resourcing		Number of medical equipment			869 (sd = 128)	
Hospital efficiency and effectiveness		Total occupied bed-days within a year			201,170 (sd = 15,847)	
**Contextual level characteristics (*n* = 10)**						
Regional economic level		Per capita GDP			71,260 (sd = 10,100)	
Medical Resource Allocation		Number of doctors			2.987 (sd = 0.127)	
		Number of general practitioners			3.965 (sd = 0.395)	
Health service system continuity		Rate of down-referral			0.010 (sd = 0.005)	
Primary health service system		Rate of family doctor contract services			0.801 (sd = 0.039)	
Government subsidy		Proportion of government subsidized income			0.189 (sd = 0.026)	

^†^Reference levels of categorical variables in the regression; UEBMI refers to Urban Employee Basic Medical Insurance; URRBMI refers to Urban Rural Resident Basic Medical Insurance; LOS refers to length of stay.

### URR of patients with MBDs

**Figure 2.**
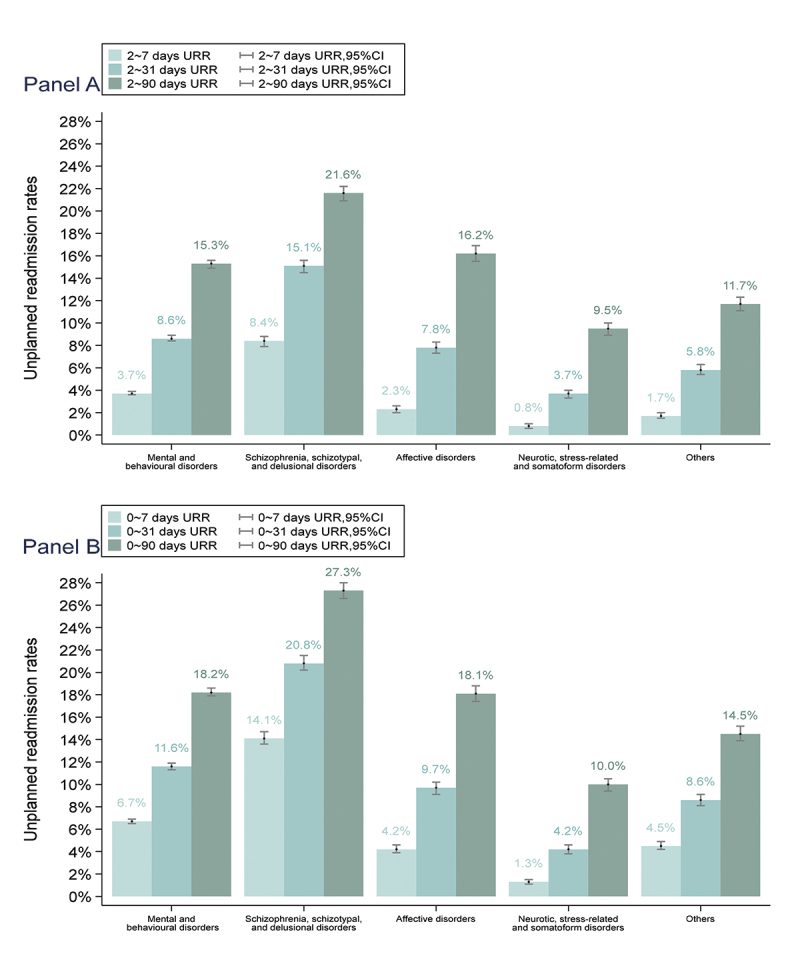
Unplanned readmission rates for mental and behavioural disorders.

Figure 2 illustrates the URR by discharge-to-readmission interval (2–7/2–31/2–90 days in Panel A and 0–7/0–31/0–90 days in Panel B) for all MBDs, including schizophrenia, affective disorders, neuroticism, and other diagnoses (statistics has been listed in Appendix Table S2). The URR for 2–7, 2–31, and 2–90 days was 3.7% (95% confidence interval (CI): 3.6–3.9%)), 8.6% (95% CI: 8.4–8.9%), and 15.3% (95% CI: 14.9–15.6%), respectively. Upon including readmissions from days 0 to 1, the URR for 0 to 7, 0 to 31, and 0 to 90 days was 6.7% (95% CI: 6.5–6.9%), 11.6% (95% CI: 11.3–11.9%), and 18.2% (95% CI: 17.9–18.6%), respectively, significantly higher than that calculated from day 2 after discharge.

(i) Mental and behavioural disorders were defined as ICD-10 codes F00-F99, schizophrenia, schizotypal, and delusional disorders as ICD-10 codes F20-F29, affective disorders as ICD-10 codes F30-F39, and neurotic, stress-related and somatoform disorders as ICD-10 codes F40-F48. (ii) The timeframe of URRs in panel A was 2–7 days, 2–31 days, and 2–90 days, respectively; the timeframe of URRs in panel B was 0–7 days, 0–31 days, and 0–90 days, respectively. (iii) The statistics has been listed in Appendix Table S3.

Subgroup analysis suggested that the URR for 2–31 days was 15.1% (95% CI: 14.5–15.6%) for schizophrenia, 7.8% (95% CI: 7.3–8.3%) for affective disorders, 3.7% (95% CI: 3.3–4.0%) for neuroticism, and 5.8% (95% CI: 5.4–6.3%) for other diagnoses.

**Figure 3.**
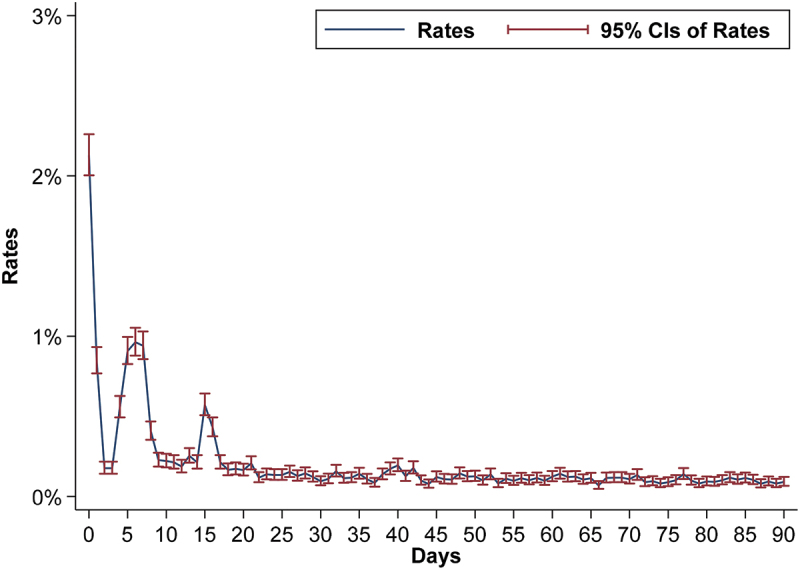
Unplanned readmission rates for mental and behavioural disorders in each of day interval over a period of 0–90 days.

Figure 3 illustrates the changes in URR across different intervals over 90 days. After discharge, the URR was high on days 0 and 1. The trends were high with short-term fluctuations (particularly from 1 to 21 days) within 90 days after discharge, before stabilising in the middle and late periods.

## Factors associated with unplanned readmissions

Table 2 summarises the results of the multilevel logistic regression analysis across three levels (univariate analyses are listed in Appendix Table S3). Model 1 indicated that most individual factors (except ethnicity and mode of admission) significantly affected the URR. Model 2 included hospital factors; individual factors demonstrated stable patterns, whereas hospital factors did not significantly affect the URR. Model 3 included contextual factors; except for the number of general practitioners, no other factor significantly affected the URR. Furthermore, both individual and hospital factors displayed consistent and reliable patterns.

**Table 2.** Analysis of influencing factors of unplanned readmissions for mental and behavioural disorders using multilevel logistic regressions.

		Model 1		Model 2		Model 3	
Variables	Subgroups	OR	95% CI	OR	95% CI	OR	95% CI
Age group	18–40	1.114	(0.946–1.312)	1.112	(0.944–1.309)	1.107	(0.940–1.304)
	41–65	1.217**	(1.018–1.453)	1.213**	(1.015–1.450)	1.209**	(1.012–1.444)
	65 or above	1.086	(0.892–1.321)	1.081	(0.888–1.316)	1.075	(0.883–1.308)
Gender	Female	0.923**	(0.855–0.997)	0.923**	(0.855–0.998)	0.924**	(0.855–0.998)
Ethnicity	Non-Han	1.032	(0.684–1.557)	1.031	(0.683–1.556)	1.029	(0.682–1.553)
Occupational status	Unemployed	1.056	(0.885–1.259)	1.057	(0.886–1.261)	1.055	(0.885–1.259)
	Non-Employed	1.266***	(1.118–1.434)	1.266***	(1.118–1.434)	1.262***	(1.114–1.429)
	Others	1.387***	(1.240–1.552)	1.388***	(1.241–1.553)	1.387***	(1.240–1.552)
Marital status	Married	0.601***	(0.541–0.666)	0.600***	(0.541–0.666)	0.600***	(0.540–0.666)
	Others	1.008	(0.896–1.134)	1.008	(0.896–1.134)	1.007	(0.896–1.133)
Medical insurance	UEBMI	1.896***	(1.620–2.220)	1.896***	(1.620–2.220)	1.894***	(1.618–2.217)
	URRBMI	1.449***	(1.255–1.672)	1.446***	(1.253–1.670)	1.447***	(1.253–1.670)
	Others	1.592***	(1.324–1.914)	1.596***	(1.328–1.918)	1.596***	(1.328–1.918)
Diagnostic group	Affective disorders	0.641***	(0.582–0.706)	0.641***	(0.582–0.707)	0.641***	(0.582–0.707)
	Neuroticism	0.548***	(0.474–0.634)	0.546***	(0.472–0.631)	0.546***	(0.472–0.632)
	Others	0.691***	(0.607–0.786)	0.688***	(0.605–0.784)	0.688***	(0.605–0.784)
Condition on admission	Clinically undetermined	0.923	(0.741–1.151)	0.922	(0.739–1.149)	0.915	(0.734–1.141)
	Unknown or no symptom	0.693***	(0.527–0.912)	0.693***	(0.527–0.912)	0.693***	(0.527–0.911)
Mode of admission	Elective admission	1.110	(0.951–1.295)	1.113	(0.954–1.300)	1.114	(0.954–1.300)
	Others	1.028	(0.631–1.673)	1.072	(0.656–1.749)	1.002	(0.613–1.638)
Mode of discharge	Transferred	1.387	(0.853–2.254)	1.385	(0.852–2.251)	1.383	(0.851–2.248)
	Discharged against medical advice	1.494***	(1.169–1.909)	1.497***	(1.172–1.913)	1.500***	(1.174–1.917)
	Others	0.560*	(0.294–1.069)	0.575*	(0.302–1.093)	0.641	(0.339–1.213)
Other diagnoses	With other psychiatric diagnoses	0.801**	(0.644–0.997)	0.803*	(0.645–1.000)	0.808*	(0.649–1.006)
	With other non-psychiatric diagnoses	0.918**	(0.844–0.999)	0.918**	(0.843–0.999)	0.919**	(0.844–1.000)
LOS	8–14 days	1.121	(0.962–1.307)	1.125	(0.965–1.312)	1.124	(0.964–1.310)
	15–30 days	1.577***	(1.352–1.839)	1.588***	(1.361–1.855)	1.593***	(1.364–1.860)
	30 days or above	1.748***	(1.449–2.109)	1.764***	(1.460–2.130)	1.772***	(1.467–2.140)
Total medical cost	Middle	0.859***	(0.773–0.953)	0.858***	(0.773–0.953)	0.855***	(0.770–0.950)
	High	0.777***	(0.665–0.908)	0.778***	(0.666–0.910)	0.776***	(0.664–0.907)
Hospital grade	Tertiary hospitals			0.792	(0.376–1.669)	0.866	(0.427–1.757)
Hospital type	Mental specialized hospital			0.809	(0.310–2.113)	0.713	(0.261–1.950)
Number of employees				0.713	(0.167–3.049)	0.475	(0.128–1.759)
Total occupied bed-days				0.926	(0.490–1.753)	0.890	(0.427–1.854)
Number of medical equipment				0.953	(0.748–1.214)	0.977	(0.766–1.247)
Total hospital revenue				1.506	(0.533–4.258)	1.920	(0.730–5.050)
Per capita GDP						1.069	(0.798–1.432)
Number of doctors						1.139	(0.898–1.446)
Number of general practitioners						0.704**	(0.501–0.988)
Rate of down-referral						1.365	(0.851–2.188)
Rate of family doctor contracts						1.315	(0.861–2.009)
Proportion of government subsidy						1.008	(0.655–1.550)
Constant		0.040***	(0.028–0.058)	0.009*	(0.000–1.608)	0.010*	(0.000–1.602)
Observations		49,352		49,352		49,352	

Details on the definitions and measurements of the independent variables are provided in Appendix Table S1; The reference group for each variable is labeled in Table 1; ***p < 0.01, **p < 0.05, *p < 0; UEBMI refers to Urban Employee Basic Medical Insurance; URRBMI refers to Urban Rural Resident Basic Medical Insurance; LOS refers to length of stay. The null model for the URR estimated that the value of the intraclass correlation coefficient was 0.219 (*p* < 0.001).

Specifically, for all MBDs, Model 3 demonstrated that a higher number of general practitioners in prefecture-level cities was associated with a lower likelihood of unplanned readmission (odds ratio (OR) = 0.704, 95% CI = 0.501–0.988). Regarding demographic and sociological factors, the risk factors for unplanned readmissions included age from 41 to 65 years, male sex, non-employed or other occupational status, unmarried status, and having medical insurance covering inpatient costs. Regarding disease-related factors, patients with affective disorders (OR = 0.641, 95% CI: 0.582–0.707), neuroticism (OR = 0.546, 95% CI: 0.472–0.632), and other psychiatric disorders (OR = 0.688, 95% CI: 0.605–0.784) were less likely to be readmitted than patients with schizophrenia. Furthermore, patients with unknown or no primary diagnostic symptoms during admission were 30.7% (OR = 0.693, 95% CI: 0.527–0.911) less likely to be readmitted than patients with symptoms. Inpatients with concurrent nonpsychiatric diagnoses (OR = 0.919, 95% CI = 0.844–1.000) were less likely to be readmitted. Regarding clinical factors, a longer LOS, lower medical costs during hospitalisation, and discharge against medical advice were associated with a greater likelihood of unplanned readmission.

## Subgroup analysis by primary diagnosis groups

**Figure 4.**
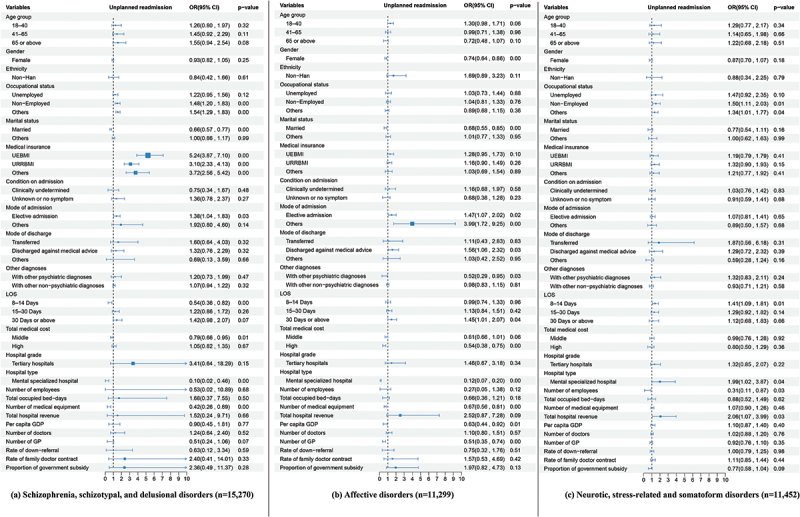
Analysis of influencing factors of unplanned readmissions for subgroups.

Forest plots in Figure 4 illustrate the results of subgroup analyses based on different diagnoses (i.e. schizophrenia, affective disorders, and neuroticism; statistics are listed in Appendix Table S4). The individual factors affecting unplanned readmissions varied across subgroups.

Three separate subgroup analyses were conducted on patients with schizophrenia, schizotypal, and delusional disorders; affective disorders; and neurotic, stress-related, and somatoform disorders. Forest plots were utilised to visually represent the results in panels (a), (b), and (c). Squares represent the odds ratio, with error bars corresponding to the 95% CI bounds, which were calculated by using the multilevel binary logistic regression models. UEBMI refers to Urban Employee Basic Medical Insurance; URRBMI refers to Urban Rural Resident Basic Medical Insurance; LOS refers to length of stay; GP refers to general practitioner. The statistics has been listed in Appendix Table S4.

Several hospital-level factors were found to have significant effects in these specific subgroups. Patients with schizophrenia and affective disorders were less likely to be readmitted to specialised mental hospitals than to general hospitals. In contrast, the likelihood increased for patients with neuroticism. Human resources served as a significant protective factor against unplanned readmission in the neuroticism subgroup. The facility size served as a significant protective factor in the schizophrenia and affective disorder subgroups. Hospital revenue served as a significant risk factor in the affective disorders and neuroticism subgroups.

In addition to the number of general practitioners, several other contextual factors exerted a significant effect. Specifically, the regional economic level served as a significant protective factor in the affective disorder subgroup. The proportion of government subsidies for public hospitals served as a protective factor at a 0.1 significance level in the neuroticism subgroup.

## Discussion

In this study, we estimated URRs among patients with MBDs across different time intervals. Additionally, we investigated the individual, hospital, and contextual factors of 2–31 days of unplanned readmissions. There were three key findings. First, the 2- to 31-day URR for total MBDs was 8.6% (95% CI: 8.4–8.9%), and disproportionately high URRs were recorded on days zero and one after discharge. Second, patients with schizophrenia had a higher 2- to 31-day URR of 15.1% (95% CI: 14.5–15.6%) compared with patients with affective disorders (7.8%, 95% CI: 7.3–8.3%) and neuroticism (3.7%, 95% CI: 3.3–4.0%). Third, individual (e.g. sex, age, marital status, occupational status, medical insurance status, diagnostic group, admission condition, mode of discharge, other diagnoses, LOS, and initial hospitalisation costs), hospital (e.g. hospital type, human resources, facility size, and revenue size), and contextual (e.g. the number of general practitioners, regional economic level, and the proportion of public hospital subsidies) factors significantly affected URRs across all MBDs and in specific subgroups, with variations among subgroups.

Across varied metrics, the URR for patients with MBDs (8.6%, 95% CI: 8.4–8.9%) from the study region in China ranked intermediate on an international scale. Zhou et al. reported that the 30-day URR of inpatients with psychiatric disorders ranged from 4.5% [5] to 43.3% [6] across six countries/regions [1]. In China, the all-cause URR within 31 days of discharge from secondary and public hospitals was 2.92% in 2019 [4]. The higher URR for patients with psychiatric conditions than for patients with general health conditions is consistent with previous findings [1,7]. Han et al. [28] reported a 16.69% 30-day readmission rate among inpatients with psychiatric disorders in Beijing, China. In contrast, Cheng et al. [39] reported a 6.0% 60-day readmission rate among patients with major depressive disorder in a Chinese mental health centre. However, these studies did not differentiate between planned and unplanned readmissions. There are differences in ‘readmission’ definitions and measurements across countries, indicating policy-driven goals. Therefore, researchers should cautiously compare intercountry and interregional data [2]. Additionally, daily URR measurements for a 90-day post-discharge period indicated higher short-term URRs, necessitating prompt follow-up for patients with psychiatric conditions [8,40,41].

Among sociodemographic characteristics, individual factors, such as age, gender, marital status, employment status, and medical insurance usage, significantly affected the URR among inpatients with psychiatric conditions. Specifically, patients aged 41–65 years, non-employed, and with partial or complete medical insurance were at a high risk of unplanned readmissions. In contrast, female sex and married status were the protective factors against unplanned readmissions. The associations of URRs with sex, marital status, and employment status were consistent with previous findings [9,10,12,19,42–44]. Likewise, the association between ages 41 to 65 years and higher URRs is consistent with a previous finding [45] but not with others [5,6,43,46]. Furthermore, some studies did not demonstrate the effect of age on short-term readmissions [28]. Inpatients with medical insurance demonstrated a higher URR than self-paying patients, particularly those covered by urban employee basic medical insurance, which requires lower co-payments. For inpatients, medical insurance mitigates economic risks and may reduce the motivation to avoid unplanned readmissions. Hospitals may have more induced demand for insured patients when medical institutes do not take action against unplanned readmissions. In addition, control strategies by insurance institutions regarding hospitalisation expenditures and LOS may result in split hospitalisation, as supported by the disproportionately high URRs at days zero and one.

Among individual-level disease factors, the primary diagnosis group, with other diagnoses, and admission condition significantly affected unplanned readmissions. Patients with schizophrenia had significantly higher URRs than patients with affective disorders and neuroticism, consistent with the findings by Lorine et al. [35]. Compared with inpatients with no other diagnosis, patients with other nonpsychiatric diagnoses had significantly lower URRs, a pattern observed in previous findings [1,19,42] but not in others [44,45]. This association may be related to a more specific other diagnostic classification [1].

Among individual-level clinical factors, longer LOS and discharge against medical advice were the risk factors for URR, whereas higher hospitalisation cost was a protective factor. Intensive treatment may have contributed to the inverse association between higher hospitalisation costs and lower URRs among inpatients with psychiatric conditions. Associations between LOS and URR remain contradictory [43,46–49]. Lin et al. [43] reported that an LOS of 5–7 days reduced readmission likelihood by 21%, whereas LOS over 15 days increased readmission likelihood by 37%. A similar pattern was observed in patients with schizophrenia, where an 8- to 14-day LOS served as a protective factor against unplanned readmissions, whereas LOS ≥ 30 days served as a risk factor at 0.1 level of significance.

Hospital factors such as hospital type, resource availability, and revenue were associated with the URR for specific diagnoses. For patients with schizophrenia and affective disorders, mental speciality hospitals were associated with low URRs, whereas an opposite association was observed for patients with neuroticism. There is a lack of consensus on the international typology of hospital types, and these findings are likely to be contingent upon their specific context [41,50]. This heterogeneity may reflect the variations in resource allocation, diagnosis, and treatment capacity of specialised and general hospitals. Human resources and hospital equipment served as significant protective factors against unplanned readmission among patients with neuroticism, schizophrenia, and affective disorders. A potential explanation is that the diagnostic and treatment capacity of medical institutes improves the quality of medical services, thereby lowering URRs [50]. Higher hospital revenue was associated with increased URRs in patients with affective disorders and neuroticism, probably indicating greater economic drivers and complex conditions. However, the mechanism underlying this association is unclear and warrants further investigation.

At the contextual level, the number of general practitioners per 1,000 people in prefecture-level cities served as a significant protective factor against unplanned readmissions in patients with MBDs. More general practitioners indicate a stronger primary care system, leading to better continuing care after discharge from inpatient and specialist services. Despite limited evidence, a strong workforce in primary care is associated with a lower incidence of readmissions [16]. Management services for severe mental disorders are pivotal in China, with general practitioners serving as the key providers [51]. They focus on continuity of care, prevention and interventional treatment, and equal access to public health services.

These findings have several policy implications. First, health systems should improve the accessibility, quality, and continuity of care [52] focusing on the number of general practitioners. Improved transitions from hospital to post-hospital care can reduce readmissions by 30–50%; transition failures are a major contributing factor to hospital readmissions [53]. Continuity in mental health services may facilitate smoother transitions for patients with complex conditions and reduce hospitalisations and readmissions [54]. Second, health administrators should incentivise hospitals to reduce avoidable readmissions using both economic and noneconomic measures, such as incorporating readmission rates into performance-based payments, penalising hospitals with higher-than-expected URRs, and publicly reporting URRs [36]. Most readmissions result from clinical deterioration and are often necessary. These incentives will ensure adequate hospital equipment and evidence-based disease management programmes to provide optimal mental health care, rather than only reducing URRs [53]. Third, the health insurance sector should regulate unreasonable medical behaviour on split admissions [55] and optimise prepaid systems to encourage hospitals to reduce URRs through bundled payments [56]. Fourth, hospitals can use risk prediction models to identify and manage high-risk patients for readmissions [57], health education, medication compliance plans, tailored discharge instructions, and timely follow-ups [58].

To the best of our knowledge, this study is among the few multicentre, large-sample empirical studies on psychiatric inpatient URRs in China. This sample spanned 99 hospitals (tertiary and secondary hospitals) and different settings (general and specialised) across 10 prefecture-level cities. Multi-center studies with large samples tend to have less bias. Moreover, this study expanded the scope of analysing risk and protective factors against unplanned readmissions, which were rarely examined before [41]. The individual, hospital, policy-related, and environmental factors jointly affected URRs among inpatients with psychiatric conditions. Additionally, establishing evidence-based readmission control policies is essential to improve the quality of medical care and reduce medical expenditure.

This study has several limitations. First, the individual-level factors were collected from the front pages of medical records, which may provide insufficient data. Additional indicators such as the clinical course, socioeconomic characteristics, social support, and health-related behaviours should be examined in the future. Although this approach would require additional questionnaires and language models to process electronic medical records, it could provide more valuable clinical process indicators. Second, despite significant differences in URRs among patients with MBDs across hospitals and regions, higher numbers of significant explanatory factors at the hospital and contextual levels remain unclear. This limitation is partly attributed to data unavailability, insufficient regional grouping of samples, and the low variability of indicators in prefecture-level cities in the same province. Despite the large sample size and multicentre design, the single regional context introduces uncertainties regarding the generalisability of our findings to other regions. Future research should address these limitations by analysing a wide range of variables. Third, this study focused only on factors affecting the 2 to 31-day URRs. The factors influencing readmissions may vary across different time intervals. Owing to space constraints, this limitation will be addressed in the future.

## Conclusions

In this study, URRs among inpatients with psychiatric conditions in China were found to be at a moderate level compared with international standards. To reduce the risks of unplanned readmissions, clinicians should identify the significant risk and protective factors. This study emphasises addressing healthcare provider-related factors, the underlying social environment, healthcare resources, and health systems that affect health and care delivery. Clinicians should assess specific sociodemographic, disease-related, and clinical characteristics to identify the determinants of unplanned readmissions among patients with MBDs.

## Supplementary Material

Appendix_clean_R3.docx
