# Supplementary material for: Factors associated with unplanned readmissions for patients with mental and behavioural disorders in China: a quantitative analysis
Source: Glob Health Action. 2025 Jan 20;17(1):2435642. doi: 10.1080/16549716.2024.2435642 (PMC11749006; doi:10.1080/16549716.2024.2435642)
Supplement: Appendix_clean_R3.docx [file ZGHA_A_2435642_SM8124.docx]

**Appendix**

Table S1 Definitions and measurements of the variables included in the analyses

| **Variables** | **Definition** |
| --- | --- |
| **31-day unplanned readmission** | The binary outcome variable was encoded as zero for not experience an unplanned readmission within 31-day or one for experience an unplanned readmission within 31-day of discharge from index admission. Readmission of 0-1 days was not considered unplanned readmission in the regression analyses. |
| **Individual-level factors** | **Demographic-sociological factors, disease characteristics, and clinical** **characteristics were included.** |
| Gender | Male and Female |
| Age | Aged 0–17, 18–40, 41–65 and 65 or above years |
| Ethnicity | Han and Non-han |
| Occupational status | Employed, Unemployed, Non-Employed (students and retired workers) and Others (freelancers, self-employed and others) |
| Marital status | Unmarried, Married and Others (widowed, divorced and others) |
| Medical insurance | Self-pay, Urban Employee Basic Medical Insurance (UEBMI), Urban Rural Resident Basic Medical Insurance (URRBMI) and Others (i.e., indigent assistance, commercial health insurance, full public pay, other social insurance and others) |
| Condition on admission of main diagnosis | Symptomatic, Clinically undetermined and Unknown or no symptom |
| Diagnostic group | Schizophrenia, schizotypal, and delusional disorders (F20-F29), Affective disorders (F30-F39), Neurotic, stress-related and somatoform disorders (F40-F48) and Others (other than the above three categories of mental diseases) |
| Mode of admission | Emergency, Elective admission and Others (includes transfers from other medical institutions and others) |
| Mode of discharge | Discharge following medical advice, Transferred following medical advice, Discharged against medical advice and Others (other than the above three ways) |
| Other diagnoses | No other diagnoses, With other psychiatric diagnoses and With other non-psychiatric diagnoses |
| Length of stay (LOS) | 0–7 days ^†^, 8–14 days, 15–30 days and 30 or above days |
| Total medical cost of previous hospitalization | The total medical cost of hospitalization includes the fee of drugs, laboratory tests, radiological examinations, and medical procedures during the hospital stay. It was divided into three groups according to the tertile points: low (less than CNY 6,244.60), middle (CNY 6,246.87 - 12,400.68), and high (CNY 12,405.00 or above). |
| **Hospital-level factors** | **The accreditation level, type, size, volumes and revenue of each hospital were included** |
| Hospital grade | Categorized into two groups: secondary hospitals and tertiary hospitals. Based on hospital functions, the public hospitals in China were classified into three grades by Health Administration, namely, primary hospitals, secondary hospitals and tertiary hospitals. |
| Hospital type | General hospital and Mental specialized hospital |
| Number of active employees | It refers to the number of hospital staff, including doctors, nurses, administrative staff, interns and other employees working in each hospital. The log value was taken in regressions. |
| Total occupied bed-days within a year | It refers to the total number of occupied bed-days occupied by discharged patients in each hospital within a year. The log value was taken in regressions. |
| Number of medical equipment with a value of ≥ CNY 10,000 | It refers to the number of medical equipment with a value of ≥ CNY 10,000 at the end of the year in each hospital. The log value was taken in regressions. |
| Total hospital revenue | It refers to the sum of all revenues in one year each hospital from the provision of medical services and other related operations over. The log value was taken in regressions. |
| **Contextual-level factors** | **Indicators reflecting economic level, health resources, continuity of health services and primary health care for each prefecture-level city were included.** |
| Per capita Gross Domestic Product (GDP) | It refers to the ratio of each prefecture-level city's GDP to its total population. It was normalized by Z-score in regressions. |
| Number of doctors | The number of practicing and assistant physicians per 1,000 population in prefecture-level cities were used. It was normalized by Z-score in regressions. |
| Number of general practitioners | The number of general practitioners per 10,000 population in prefecture-level city were used. It was normalized by Z-score in regressions. |
| Rate of down-referral | It refers to the percentage of hospitalized patients referred from hospitals to community health systems in prefecture-level city and captures continuity of health services. It was normalized by Z-score in regressions. |
| Rate of family doctor contract services | It refers to the proportion of key contracted population groups with family doctor services to the total key population in prefecture-level cities. It was normalized by Z-score in regressions. |
| Proportion of government subsidized income in public hospitals | It refers to the proportion of government subsidized income to total income in public hospitals. It was normalized by Z-score in regressions. |

Table S2 Unplanned readmission rates for mental and behavioural disorders

|  | Time interval | URR | Time interval | URR |
| --- | --- | --- | --- | --- |
| Total mental and behavioural disorders | 2–7 days | 0.037(0.036-0.039) | 0–7 days | 0.067(0.065-0.069) |
|  | 2–31 days | 0.086(0.084-0.089) | 0–31 days | 0.116(0.113-0.119) |
|  | 2–90 days | 0.153(0.149-0.156) | 0–90 days | 0.182(0.179-0.186) |
| Schizophrenia, schizotypal, and delusional disorders | 2–7 days | 0.084(0.079-0.088) | 0–7 days | 0.141(0.136-0.147) |
|  | 2–31 days | 0.151(0.145-0.156) | 0–31 days | 0.208(0.202-0.215) |
|  | 2–90 days | 0.216(0.209-0.222) | 0–90 days | 0.273(0.266-0.280) |
| Affective disorders | 2–7 days | 0.023(0.020-0.026) | 0–7 days | 0.042(0.039-0.046) |
|  | 2–31 days | 0.078(0.073-0.083) | 0–31 days | 0.097(0.091-0.102) |
|  | 2–90 days | 0.162(0.155-0.169) | 0–90 days | 0.181(0.174-0.188) |
| Neurotic, stress-related and somatoform disorders | 2–7 days | 0.008(0.006-0.010) | 0–7 days | 0.013(0.011-0.015) |
|  | 2–31 days | 0.037(0.033-0.040) | 0–31 days | 0.042(0.038-0.046) |
|  | 2–90 days | 0.095(0.089-0.100) | 0–90 days | 0.100(0.094-0.105) |
| Others | 2–7 days | 0.017(0.015-0.020) | 0–7 days | 0.045(0.042-0.049) |
|  | 2–31 days | 0.058(0.054-0.063) | 0–31 days | 0.086(0.081-0.091) |
|  | 2–90 days | 0.117(0.111-0.123) | 0–90 days | 0.145(0.139-0.152) |

Note: URR refers to unplanned readmission rate.

Table S3 Univariate analysis of unplanned readmission and related variables (chi-square value, p-value)

|  | Overall (%)  (n=49,352) | Schizophrenia, schizotypal, and delusional disorders (%)  (n=15,270) | Affective disorders (%)  (n= 11,299) | Neurotic, stress-related and somatoform disorders (%)  (n=11,452) |
| --- | --- | --- | --- | --- |
| **Individual level factors** | | | | |
| Gender | 180.0113*** | 68.7744*** | 38.4923*** | 1.4629 |
| Age | 171.3574*** | 255.8375*** | 33.0843*** | 10.7758** |
| Marital status | 566.6526*** | 185.7823*** | 34.0610*** | 15.3395*** |
| Ethnicity | 1.5362 | 0.9356 | 0.0747 | 1.2488 |
| Occupational status | 167.2391*** | 173.7480 *** | 12.8497*** | 13.2527*** |
| Medical insurance | 583.2703*** | 655.9527 *** | 11.5340*** | 5.2853 |
| Diagnostic group | 1300*** |  |  |  |
| Condition on admission of main diagnosis | 126.3002*** | 63.8172 *** | 3.5611 | 4.3103 |
| Mode of admission | 804.1436*** | 611.4842 *** | 7.4956** | 2.6583 |
| Mode of discharge | 229.5506*** | 337.4419 *** | 4.2542 | 5.4814 |
| Other diagnoses | 818.6395*** | 280.2116 *** | 38.5689 *** | 6.4159** |
| LOS | 1000*** | 952.5791 *** | 1.5373 | 25.3018*** |
| Total medical cost of initial hospitalization | 268.9236*** | 821.1285 *** | 51.7611*** | 3.8851 |
| **Between hospitals** | 5541.42*** | 3593.36*** | 345.18*** | 124.12*** |
| **Between prefecture level cities** | 621.7926*** | 735.7689*** | 36.7919*** | 6.7570 |

Note: Differences between hospitals were analysed using univariate logistic regression, differences between groups in other categorical variables were performed using chi-square tests; *** p<0.01, ** p<0.05, * p<0.1; LOS refers to Length of stay.

Table S4 Analysis of influencing factors of unplanned readmissions for subgroup of disorders using multilevel logistic regressions

|  |  | Schizophrenia, schizotypal, and delusional disorders | | Affective Disorders | | Neurotic, stress-related and somatoform disorders | |
| --- | --- | --- | --- | --- | --- | --- | --- |
| Variables | Subgroup | OR | 95%CI | OR | 95%CI | OR | 95%CI |
| Age group | 18–40 | 1.258 | (0.803 - 1.970) | 1.297* | (0.985 - 1.709) | 1.291 | (0.767 - 2.172) |
|  | 41–65 | 1.451 | (0.918 - 2.292) | 0.992 | (0.714 - 1.378) | 1.135 | (0.650 - 1.980) |
|  | 65 or above | 1.546* | (0.942 - 2.535) | 0.717 | (0.482 - 1.067) | 1.216 | (0.677 - 2.183) |
| Gender | Female | 0.929 | (0.821 - 1.052) | 0.745*** | (0.642 - 0.865) | 0.866 | (0.699 - 1.071) |
| Ethnicity | Non-Han | 0.836 | (0.421 - 1.663) | 1.693 | (0.887 - 3.232) | 0.880 | (0.344 - 2.248) |
| Occupational status | Unemployed | 1.217 | (0.947 - 1.562) | 1.026 | (0.730 - 1.441) | 1.474 | (0.923 - 2.355) |
|  | Non-Employed | 1.484*** | (1.201 - 1.832) | 1.039 | (0.813 - 1.327) | 1.505*** | (1.113 - 2.034) |
|  | Others | 1.538*** | (1.295 - 1.827) | 0.886 | (0.684 - 1.147) | 1.338** | (1.014 - 1.767) |
| Marital status | Married | 0.663*** | (0.573 - 0.767) | 0.684*** | (0.550 - 0.852) | 0.774 | (0.541 - 1.107) |
|  | Others | 1.001 | (0.857 - 1.170) | 1.009 | (0.766 - 1.329) | 1.002 | (0.616 - 1.631) |
| Medical insurance | UEBMI | 5.245*** | (3.872 - 7.104) | 1.283 | (0.953 - 1.727) | 1.189 | (0.788 - 1.794) |
|  | URRBMI | 3.103*** | (2.329 - 4.135) | 1.157 | (0.900 - 1.489) | 1.320 | (0.902 - 1.932) |
|  | Others | 3.723*** | (2.559 - 5.415) | 1.028 | (0.687 - 1.540) | 1.213 | (0.767 - 1.919) |
| Condition on admission | Clinically undetermined | 0.753 | (0.339 - 1.669) | 1.160 | (0.684 - 1.966) | 1.035 | (0.756 - 1.415) |
|  | Unknown or no symptom | 1.362 | (0.784 - 2.366) | 0.680 | (0.362 - 1.279) | 0.912 | (0.588 - 1.414) |
| Mode of admission | Elective admission | 1.375** | (1.036 - 1.826) | 1.470** | (1.070 - 2.020) | 1.067 | (0.806 - 1.413) |
|  | Others | 1.922 | (0.802 - 4.604) | 3.986*** | (1.718 - 9.249) | 0.886 | (0.500 - 1.569) |
| Mode of discharge | Transferred | 1.601 | (0.636 - 4.027) | 1.108 | (0.434 - 2.827) | 1.867 | (0.564 - 6.182) |
|  | Discharged against medical advice | 1.322 | (0.761 - 2.295) | 1.565** | (1.056 - 2.320) | 1.295 | (0.721 - 2.324) |
|  | Others | 0.692 | (0.134 - 3.586) | 1.027 | (0.419 - 2.519) | 0.592 | (0.282 - 1.242) |
| Other diagnoses | Psychiatric other diagnoses | 1.204 | (0.727 - 1.994) | 0.522** | (0.287 - 0.950) | 1.322 | (0.828 - 2.111) |
|  | Non-psychiatric other diagnoses | 1.068 | (0.939 - 1.216) | 0.980 | (0.835 - 1.151) | 0.929 | (0.714 - 1.208) |
| LOS | 8–14 days | 0.545*** | (0.360 - 0.825) | 0.993 | (0.742 - 1.330) | 1.407*** | (1.095 - 1.808) |
|  | 15–30 days | 1.219 | (0.865 - 1.718) | 1.127 | (0.843 - 1.507) | 1.293 | (0.915 - 1.825) |
|  | 30 days or above | 1.422* | (0.977 - 2.069) | 1.448** | (1.013 - 2.069) | 1.118 | (0.682 - 1.835) |
| Total medical cost | Middle | 0.793** | (0.663 - 0.948) | 0.815* | (0.659 - 1.006) | 0.987 | (0.757 - 1.285) |
|  | High | 1.055 | (0.824 - 1.351) | 0.537*** | (0.383 - 0.753) | 0.805 | (0.503 - 1.287) |
| Hospital grade | Tertiary hospitals | 3.414 | (0.637 - 18.287) | 1.464 | (0.674 - 3.180) | 1.324 | (0.849 - 2.066) |
| Hospital type | Mental specialized hospital | 0.102*** | (0.023 - 0.459) | 0.117*** | (0.070 - 0.197) | 1.989** | (1.022 - 3.868) |
| Number of employees |  | 0.527 | (0.025 - 10.891) | 0.267 | (0.052 - 1.381) | 0.311** | (0.111 - 0.873) |
| Total occupied bed-days |  | 1.676 | (0.372 - 7.553) | 0.663 | (0.363 - 1.213) | 0.877 | (0.518 - 1.485) |
| Number of medical equipment |  | 0.425*** | (0.262 - 0.689) | 0.671*** | (0.557 - 0.809) | 1.067 | (0.900 - 1.265) |
| Total hospital revenue |  | 1.518 | (0.237 - 9.712) | 2.518* | (0.870 - 7.284) | 2.064** | (1.067 - 3.990) |
| Per capita GDP |  | 0.900 | (0.448 - 1.808) | 0.634** | (0.439 - 0.916) | 1.105 | (0.873 - 1.400) |
| Number of doctors |  | 1.240 | (0.642 - 2.396) | 1.098 | (0.797 - 1.514) | 1.024 | (0.878 - 1.195) |
| Number of general practitioners |  | 0.508* | (0.244 - 1.056) | 0.508*** | (0.348 - 0.741) | 0.917 | (0.765 - 1.099) |
| Rate of down-referral |  | 0.631 | (0.119 - 3.340) | 0.750 | (0.320 - 1.758) | 0.997 | (0.793 - 1.254) |
| Rate of family doctor contracts |  | 2.397 | (0.410 - 14.008) | 1.571 | (0.526 - 4.693) | 1.110 | (0.854 - 1.442) |
| Proportion of government subsidy |  | 2.362 | (0.491 - 11.369) | 1.968 | (0.819 - 4.728) | 0.774* | (0.578 - 1.037) |
| Constant |  | 0.002 | (0.000 - 63.316) | 15.632 | (0.449 - 544.696) | 0.021** | (0.001 - 0.591) |
| Observations |  | 15,270 |  | 11,299 |  | 11,452 |  |

Note: Details on the definitions and measurements of the independent variables are provided in Appendix Table S1; The reference group for each variable is labeled in Table 1; *** p<0.01, ** p<0.05, * p<0.1; UEBMI refers to Urban Employee Basic Medical Insurance; URRBMI refers to Urban Rural Resident Basic Medical Insurance; LOS refers to Length of stay.
